# Supplementary material for: Structure-guided inhibition of the cancer DNA-mutating enzyme APOBEC3A
Source: Nat Commun. 2023 Oct 11;14:6382. doi: 10.1038/s41467-023-42174-w (PMC10567711; doi:10.1038/s41467-023-42174-w)
Supplement: Supplementary file 1 — Supplementary Information [file 41467_2023_42174_MOESM1_ESM.pdf]

SUPPORTING INFORMATION for

Structure-guided inhibition of the cancer DNA-mutating enzyme APOBEC3A

Stefan Harjes[1], Harikrishnan M. Kurup[1], Amanda E. Rieffer[2], Maitsetseg Bayarjargal[1,3], Jana Filitcheva[1], Yongdong Su[1,4], Tracy K. Hale[1], Vyacheslav V. Filichev*[1,5], Elena Harjes*[1,5], Reuben S. Harris*[6,7] and Geoffrey B. Jameson*[1,5]

[1] School of Natural Sciences, Massey University, Palmerston North, New Zealand

[2] Department of Biochemistry, Molecular Biology, and Biophysics, University of Minnesota–Twin Cities, Minneapolis, MN, USA

[3] Current address: Department of Biochemistry, University of Washington, Seattle, WA, USA

[4] Current address: Department of Pediatrics, Emory University School of Medicine, and the Aflac Cancer and Blood Disorders Center, Children's Healthcare of Atlanta, Atlanta, GA, USA

[5] Maurice Wilkins Centre for Molecular Biodiscovery, University of Auckland, Auckland, New Zealand

[6] Department of Biochemistry and Structural Biology, University of Texas Health San Antonio, San Antonio, TX 78023, USA

[7] Howard Hughes Medical Institute, University of Texas Health San Antonio, San Antonio, TX 78023, USA

*****Correspondence: g.b.jameson@massey.ac.nz, e.harjes@massey.ac.nz, v.filichev@massey.ac.nz, rsh@uthscsa.edu

Key words: antimutagenesis; APOBEC3A; cancer; DNA mutagenesis; hairpin DNA inhibitor; tumor evolvability

Supporting Information: Supplementary analysis and discussion, Tables S1-S4, Figures S1-S10

Supplementary analysis and discussion

Isothermal titration calorimetric results

Isothermal titration calorimetry (ITC) measurements were conducted for the binding of TTFdZ-hairpin and PS-TTFdZ-hairpin to A3A-E72A (**Supplementary Fig. S4a,b**). Binding was substantially exothermic but offset by an unfavorable entropy (**Supplementary Table S3**). At first sight this is counter-intuitive, as the hairpin is conformationally much less flexible than linear DNA; however, the negative entropy of binding reflects changes in dynamics of the protein and especially of the extended loop-3 that in substrate-free structures is highly mobile. The dissociation constants K_d cannot be compared with K_m results, as the latter is on active enzyme that offers greater hydrogen-bonding potential than the former, as the former is on inactive enzyme A3A-E72A; additionally, to ameliorate problems of aggregation of protein due to the much higher concentrations required for ITC compared to enzymatic assays bovine serum albumin was present (30 mg/ml), as well as choline acetate (50 mM).

X-Ray crystallographic results

We obtained six structures, *i.e.*, A3A-E72A with TTC-hairpin with and without zinc, ATTC-hairpin, CTTC-hairpin, and two structures of wildtype A3A with TTFdZ-hairpin. Notwithstanding full, partial or zero occupancy of the Zn^{2+} site, all structures share a common binding of residues –2, –1, 0 and +1 to A3A-E72A, as highlighted in **Fig. 2b-e**. The nucleophilic water/hydroxide expected to be bound to the Zn^{2+} center is replaced by Cl^- in these structures and others of A3A-E72A, which all feature NaCl in the crystallization medium[40].

All structures are approximately isomorphous in space group $P2_1$. The packing of molecules in the unit cell is shown in cartoon form in **Supplementary Fig. S2**, highlighting differences in interactions between bases of hairpins for different hairpins All structures of A3A-E72A show a chloride in the active-site cavity, coordinating to the Zn^{2+}, when the latter is present. In addition to this chloride, there is a water molecule that hydrogen bonds to the chloride ion, to N3 of the cytosine at position 0, and to the main chain N of residue 72. This Cl^- and H_2O occupy, approximately, likely positions of the carboxylate oxygens of Glu72 of wildtype A3A (and also the structure of the catalytic C-terminal domain of A3G[57]). For structures of A3A, A3B and A3G where the general acid-base glutamate/glutamic acid has been mutated to alanine, this pair of atoms (Cl^- and H_2O) is ubiquitous in electron density maps of all A3A,

A3B and A3G enzymes, if not always included in the structural model structures of these enzymes[18]. For the structure of A3G with the Glu-->Ala mutation and substrate bound, a water molecule is reported at the site[58].

Consistent across all structures of A3 with single-stranded DNA oligonucleotide there is a tight turn to project substrate (or inhibitor) at position 0 into the active site. This is accomplished with non-standard torsion angles for the phosphate groups, such that phosphate...phosphate distances (as P…P) range from 6.2-7.3 Å for all nucleotides except for the phosphate group that links nucleotides at positions 0 and +1, where it shortens by more than an Ångstrom to 5.1-5.6 Å. The torsion angle for residues in the stem of the hairpin, ^{n}C3′-^{n}O3′-$^{(n+1)}$P-$^{(n+1)}$O5′ and ^{n}O4′-^{n}C4′-^{n}C5′-^{n}O5′, generally lie in the expected range for B-conformation DNA, respectively, ~-90° and –70°·. Ultra-high resolution crystal structures (PDB ID 3u89 and 2be3)[59,60] of self-complementary 12-mer, ostensibly B-DNA, reveal considerable conformational flexibility and crystallographic disorder in the dodecamer with torsion angles well outside the canonical values for B-DNA. For residues in the loop (n = –2, –1, 0), the torsion angles ^{n}C3′-^{n}O3′-$^{(n+1)}$P-$^{(n+1)}$O5′ are respectively approximately, –160°, +110° and 60°, values substantially different to B-DNA.

For the phosphate linking positions -1 and 0, this amounts to an eclipsed conformation for the torsion angle C3′-O3′-P-O1. For residues 0 and +1 in three-membered loops, the torsion angles ^{n}O4′-^{n}C4′-^{n}C5′-^{n}O5′ of ~176° also differ substantially from those generally expected for B-DNA and found in the stem of ~-70°. Essentially the conformation changes from staggered to *trans*. For the four-membered loops the residue at –3 is flipped out and in order for the C-G pair at –4 and +1 to hydrogen bond, the torsion angle ^{n}O4′-^{n}C4′-^{n}C5′-^{n}O5′ at residue +1 is ~+90°. The torsion angle ^{n}P-^{n}O5′-^{n}C5′-^{n}C4′ decreases from ~+170° in the stem and positions -2 and -1 to ~+120° at position 0.

All 2′-deoxyribose units adopt the expected C2′-endo conformation, with C5′-C4′-C3′-O3′ torsions angles in the range 120-150° (lower values generally associated with pyrimidine nucleobases and higher values with purine nucleobases, as previously observed[59,60]) except those at positions –4 and –3 in structures with four-membered loops, where to accommodate the flipped-out residue at -3, this torsion angle is ~85°, a value characteristic of A-DNA.[61]

Our assignment of nitrogen atoms to the positions in the ring of His29 is based on the following interactions. His29-Nδ_1 (amine tautomer) forms a semi-salt bridge with the phosphate oxygen

at the 3′ position of C^0, and also forms a sub-optimal hydrogen bond to deoxyribose O4′ of G^{+1}. An alternative assignment of orientation of His29 to form a canonical hydrogen bond between His29-Nδ_1 and O2 of T^{-2} would require movement of His29 into a sterically less favourable conformation, as well as destroying the bifurcated hydrogen bond that locks in the sharp-turn. Moreover, the semi-salt bridge described above is much stronger than a canonical hydrogen bond between neutral parties in alternative assignment. In the highest resolution structure featuring the ATTC-hairpin, a well-defined water is observed off the assigned Nε_2 atom at 2.80 Å, which further confirms the assignment of atoms in His29.

Inter alia, O3¢ at position -1 forms a hydrogen bond to Nδ_1 of His29. His29-Nδ1 makes contact (3.4 Å) with ribose O4′ of guanine +1 and to phosphate O of cytosine 0, which in turns supports base pairing of G^{+1} with C^{-3}. His29-Cδ2 makes a non-classical hydrogen bond with carbonyl oxygen O2 of thymine–2; and His29-Nε2 hydrogen bonds to a well-defined water. Finally, His29 pi stacks with G^{+1}, possibly as cation-π if His29 is protonated. Arg28 forms a cation-π interaction with T^{-2}.

Altogether, the A3A-hairpin interaction is provided by about 40 protein atoms and about 40 nucleotide atoms, highlighted as spheres with associated residues as sticks. Those are in van der Waals or hydrogen bonding contact at a tight threshold of 3.5 Å. A space-filling representation (**Supplementary Fig. S3a**) further highlights the key role of His29, Arg28 and Loop-3 in controlling the conformation of the loop and determining preference of A3A for purines in +1 and pyrimidines in –2 positions and for hairpin oligonucleotides over linear ssDNA.

Structure of A3A-E72A (with Zn^{2+}) bound to TTC-hairpin

This structure was determined to a resolution of 2.22 Å. The Zn^{2+} ion is present in only about half occupancy. The chloride ion is present in full occupancy and is bound to the Zn^{2+}, if Zn^{2+} is present. The $T^{-1}C^0N^{+1}$ (where N is G, T or A) moiety adopts a conformation very similar to that seen for linear oligo binding in one structure to A3A (PDB: 5keg[19]), but for binding in another A3A structure (5sww[18]) only the TC moiety closely aligns. All residues of the hairpin are observed, and the thymines at each foot of the stem hydrogen bond to each other (**Supplementary Fig. S2a-c**). In the previous structure of A3A-E72A (5keg) the cysteine 171 was mutated to alanine, most likely to prevent possible dimerization but in our structure Cys171

is present in reduced form. The interaction of TTC-hairpin with A3A-E72A is shown in **Supplementary Fig. 4c**.

Structure of A3A-E72A (without Zn^{2+}) bound to TTC-hairpin

This structure was determined to a resolution of 2.10 Å. The Zn^{2+} ion is entirely absent. Its absence is not an artefact of synchrotron radiation-induced damage, as an in-house data set from a crystal from the same drop also showed an absence of Zn^{2}. The chloride ion is present in full occupancy and located in the same position as in the Zn^{2+}-containing structures. The hairpin oligonucleotide superimposes closely on that for the (partially) Zn^{2+}-containing structure, A3A-E72A/TTC-hairpin. However, as illustrated in **Supplementary Fig. S3a, b**, there are substantial changes in positions of the otherwise Zn^{2+}-coordinating residues, as they seek to minimise repulsion. In particular, the loop bearing Zn^{2+}-binding Cys101 and Cyts106 flips to move these Cys away from each other and the other otherwise Zn^{2+}-binding ligand His70.

Structure of A3A-E72A (with Zn^{2+}) bound to ATTC-hairpin

The structure of A3A with a potentially 6-base-pair stem and a 4-membered loop was determined to 1.91-Å resolution. The T^{-2}TC motif binds the same as for the three-membered loop (**Fig. 2b**, **c**). However, the residue at −3 is flipped out, such that the cytosine at the −4 position hydrogen-bonds with little distortion to the guanine at +1 and the stem duplex is in register with substrates bearing just three residues in the loop (**Fig. 2b**,**c**). The chloride ion and water molecule noted earlier are also present in this structure. By virtue of being the structure with highest resolution, more water molecules are observed here than in other structures. The interaction of ATTC-hairpin with A3A-E72A is shown in **Supplementary Fig. S4**. The packing of molecules is shown in **Supplementary Fig. S2d**,**e**. The low-resolution (3.15 Å) structure of A3A-E72A with CTTC-hairpin largely confirms this structure (**Fig. 2a**, **b**).

Whereas in structures with TTC-hairpin, which forms four canonical Watson-Crick base pairs and a thymine base pair, ATTC-hairpin has the potential to form an additional base-pair for a total of six Watson-Crick base pairs. However, five base pairs appear to define a “goldilocks” crystal packing. Thus, for ATTC-hairpin, in the crystalline state, there are only five base pairs and the last guanine ($^{+6}$G) interacts loosely with the $^{-8}C^{+5}$G pair of the second molecule in the

asymmetric unit (**Supplementary Fig. S2d, e**). The cytosine at position –9 is not observed and is presumably flipped out and conformationally flexible.

Structure of A3A-E72A (with Zn^{2+}) bound to CTTC-hairpin

Although this structure was determined to only 3.15 Å, the conformation of the oligonucleotide is well defined for residues –4 to +4. The shorter stem is less well defined, in part because four base pairs, one of which is an AT pair, do not confer high stability and in part because crystal packing is less effective where residues at the 5' and 3' ends of CTTC-hairpin base-stack weakly to the basal residues of the stem of the other A3A/CTTC-hairpin complex in the asymmetric unit, in contrast to the TTC- and ATTC-hairpins molecules (**Fig. 2a, b**). The 2′-deoxyribose-phosphate backbone of the loops is essentially identical to that for ATTC-hairpin, although the orientation of the flipped-out nucleobase at position –3 is possibly different. In both the ATTC- and CTTC-hairpin structures, this flipped-our nucleobase is very much more mobile than adjacent nucleobases, as evidenced by markedly higher atomic displacement parameters (*B*-values). In one subunit, the hairpin can be traced from –6 to +4 (end residue –7 not visible) and is superimposable on that for corresponding residues for ATTC-hairpin. However, in the other subunit, electron density for the hairpin is traceable from positions +6 to –4 (relative to the expected conformation and threading of the hairpin analogous to that observed in the other subunit for all other hairpins). This leads to the uncomfortable conclusion that in order to maintain crystal packing contacts, this hairpin is threaded differently so that residues GAC**C**, rather than the expected CTT**C**, form the loop; that is, there is a three-residue shift. Relevant to this is that CCC, the preferred substrate of A3G, is a substrate of A3A, although a much poorer substrate than the preferred substrate TTC of A3A and A3B. Whereas in the expected threading a $C^{-4}G^{+1}$ pair sits at the top of the stem, in the unexpected threading it is a $C^{-7}T^{-2}$ pair (**Fig. 2a** numbering).

Amongst the various structures of A3A, there is always slight variations in the position of the B subunit (and its hairpin) relative to the A subunit, as seen in **Supplementary Fig. S1**.

Structure of wildtype A3A (with Zn^{2+}) bound to TTFdZ-hairpin

This complex has been determined in two slightly different crystal forms of low isomorphism to 2.80 and 2.94 Å resolution (**Supplementary Table S2**). The molecules of the two forms and

molecules A and B of the asymmetric unit of a given form are, however, closely isostructural (**Supplementary Fig. S1a, b**).

Table S1 | List of oligos synthesized.

Name	DNA sequence,a) 5'→3'	ε260 (/L·mol-1·cm-1)	Retention time (/min)b)	ESI-MS [/Da] found/calculated
PS-TTC-hairpin [a)]	$G_{PS}C_{PS}G_{PS}C_{PS}T_{PS}T_{PS}C_{PS}G_{PS}C_{PS}G_{PS}C$	-	-	-
TTFdZ-hairpin	GCGCTT**FdZ**GCGC	94800	14.3	3311.5/3311.6
TTFdZ-hairpin (used for crystallization)	TGCGCTT**FdZ**GCGCT	112,200	14.1	3919.6/3918.7
PS-TTFdZ-hairpin	$G_{PS}C_{PS}G_{PS}C_{PS}T_{PS}T_{PS}\mathbf{FdZ}_{PS}G_{PS}C_{PS}G_{PS}C$	94,800	27.6[c)]	3472.3/3472.7
PS-TTdZ-hairpin	$G_{PS}C_{PS}G_{PS}C_{PS}T_{PS}T_{PS}\mathbf{dZ}_{PS}G_{PS}C_{PS}G_{PS}C$	94,800	17.7	3454.3/3454.7
PS-TTT-hairpin	$G_{PS}C_{PS}G_{PS}C_{PS}T_{PS}T_{PS}T_{PS}G_{PS}C_{PS}G_{PS}C$	101,700	17.8	3482.4/3482.3
PS-FdZ-linear	$A_{PS}T_{PS}T_{PS}T_{PS}\mathbf{FdZ}_{PS}A_{PS}T_{PS}T_{PS}T$	84,800	19.3	2810.2/2802.3
PS-dZ-linear	$A_{PS}T_{PS}T_{PS}T_{PS}\mathbf{dZ}_{PS}A_{PS}T_{PS}T_{PS}T$	84,800	19.2	2791.2/2790.3
PS-TTFdZ-hairpin- FAM	$G_{PS}C_{PS}G_{PS}C_{PS}T_{PS}T_{PS}\mathbf{FdZ}_{PS}G_{PS}C_{PS}G_{PS}C_{PS}$/FAM	115,760	29.6[c)]	4138.4/4139.3
PS-TTdZ-hairpin-FAM	$G_{PS}C_{PS}G_{PS}C_{PS}T_{PS}T_{PS}\mathbf{dZ}_{PS}G_{PS}C_{PS}G_{PS}C_{PS}$/FAM	115,960	30.2[c)]	4120.4/4119.3

a) PS refers to phosphorothioate linkage. This particular oligo was used as validated and supplied by IDT.

b) Reverse-phase HPLC, except where otherwise flagged.

c) Ion-exchange HPLC.

Table S2 | Crystal data, data collection and structure refinement details.

**Protein**	**A3A-E72A TTC-hairpin**	**A3A-E72 (no Zn2+) TTC-hairpin**	**A3A-E72A ATTC-hairpin**	**A3A-E72A CTTC-hairpin**	**A3A-wt/TTFdZ-hairpin (form 1)**	**A3A-wt TTFdZ-hairpin (form 2)**
Ligand	5'T(GC)$_2$TTC(GC)$_2$T	5'T(GC)$_2$TTC(GC)$_2$T	5'C$_3$ATCATTCGATG$_3$	5'GC$_2$GACCTTCGTCG$_2$G	5'T(GC)$_2$TTFdZ(GC)$_2$T	5'T(GC)$_2$TTFdZ(GC)$_2$T
PDB ID	8FIM	8FIL	8FIK		8FIJ	8FII
**Crystal data**						
Beamline (Temp.)	MX2 (100 K)	MX2 (100 K)	MX2 (100 K)	MX2 (100 K)	MX2 (100 K)	MX2 (100 K)
dataset #	#131	#250r	#84	#231	#339	#106+#108
a (/Å)	53.92	54.75	55.00	53.35	49.94	53.32
b (/Å)	56.9	57.35	57.23	56.74	56.37	57.20
c (/Å)	91.55	93.74	92.75	93.27	90.11	91.42
β (/°)	103.6	105.8	106.5	106.2	104.0	99.4
Crystal habit	Flattened needle	Thin plate	Flattened needle	Flattened needle	Flattened needle <1x2x5 mm	Flattened needle <1x2x5 mm
Crystal system	Monoclinic	Monoclinic	Monoclinic	Monoclinic	Monoclinic	Monoclinic
Space group	$P\,2_1$	$P\,2_1$	$P\,2_1$	$P\,2_1$	$P\,2_1$	$P\,2_1$
Z'	2	2	2	2	2	2
**Data collection**	**MX2 (Australian Synchrotron) – microfocus 10x5 mm**					
Resolution [highest]	47.94-2.22 [2.29-2.22]	40.94-2.01 [2.06-2.01]	48.13-1.91 [1.96-1.91]	47.93-3.15 [3.36-3.15]	48.24-2.80[2.95-2.80]	49.05-2.94 [3.12-2.94]
# reflections	89 061 [6814]	102 081 [6311]	142 158 [8859]	31 403 [4962]	39 433 [5461]	45 891 [7086]
# unique reflns	26 243 [2098]	36 510 [2435]	42 309 [2706]	9310 [1573]	11 861 [1629]	11 619 [1817]
# reflns for Rfree	1309	1827	2103	520	623	567
Redundancy	3.4 [3.2]	2.8 [2.6]	3.4 [3.3]	3.4 [3.2]	3.3 [3.4]	3.9 [3.9]
Completeness	97.3 [85.1]	97.8 [88.3]	98.4 [94.2]	98.3 [92.9]	97.8 [92.8]	99.0 [97.1]
R_{merge}	0.092 [1.535]	0.113 [1.294]	0.090 [0.913]	0.342 [1.158]	0.183 [1.157]	0.166 [0.583]
R_{pim}	0.059 [0.979]	0.081 [0.955]	0.057 [0.589]	0.218 [0.769]	0.118 [0.735]	0.094 [0.338]
$CC_{1/2}$	0.995 [0.332]	0.992 [0.188]	0.996 [0.471]	0.962 [0.621]	0.979 [0.466]	0.985 [0.918]
$I/\sigma(I)$	6.2 [0.6]	6.1 [0.7]	7.5 [1.0]	2.8 [1.0]	4.5 [0.8]	5.8 [2.2]
Mosaicity (/°)	0.37	0.15	0.08	0.34	0.28	0.33
Wilson B (/Å^2)	51	38.2	26.3	52.8	57	42.3
**Structure refinement**						
Resolution [highest]	47.94-2.22 [2.28-2.22]	40.94-2.01 [2.06-2.01]	48.17-1.91 [1.96-1.91]	47.98-3.15 [3.23-3.15]	48.29-2.80 [2.87-2.80]	48.35 [2.94] [3.02-2.94]
R_{work}	0.238 [0.372]	0.231 [0.360]	0.201 [0.0.308]	0.304 [0.433]	0.258 [0.356]	0.254 [0.376]
R_{free}	0.277 [0.419]	0.266 [0.365]	0.233 [0.302]	0.350 [0.438]	0.318 [0.432]	0.310 [0.367]
# atoms (total)	3657	3715	3937	3392	3362	3377
# protein atoms	3040	3040	3111	2926	2914	2893
# nucleotide atoms	520	520	614	447	164+240	224+224
# other atoms	2×I6P, 2×(½) Zn^{2+}, 2×Cl^-, 1xPO_4^{3-}	2×I6P, 2×Cl^-, no Zn^{2+}	3×PO_4^{3-},[a] 1×I6P, 2×Zn^{2+}, 2×Cl^-	3×PO_4,[a] 2×Zn^{2+}, 2×Cl^-	6×PO_4^{3-},[a] 2×Zn^{2+}	7×PO_4^{3-},[a] 2×Zn^{2+}
# water molecules	16	81	157	0	2	0
Mean B (/Å^2)	57.2	43.9	34.8	59.9	50.2	61
Rmsd bond distances (/Å)	0.006	0.008	0.010	0.005	0.008	0.007
Rmsd bond angles (/°)	1.58	1.84	1.90	1.26	1.65	1.57
Ramachandran (COOT) OK/OK-ish/Bad (/%)	93.5/5.2/1.4	93.8/5.3/0.9	94.4/5.0/0.6	92.2/6.8/1.1	96.6/2.8/0.6	97.7/2.9/0.0

[a] Likely a partially occupied and somewhat disordered I6P (inositol hexaphosphate).

Table S3 | ITC data for titration of A3A-E72A with TTC- or PS-TTC-hairpin[a]

Experiment	*n*	*K* [10^5/*M*]	Δ*H* [kcal/mol]	Δ*S* [cal/mol/K]
PO-TTC-hairpin	$1.08 \pm 5 \times 10^{-3}$	8.7 ± 0.6	-11.9 ± 0.07	-12.6
PO-TTC-hairpin	$1.03 \pm 5 \times 10^{-3}$	11.3 ± 0.6	-12.6 ± 0.05	-14.7
PO-TTC-hairpin	$1.08 \pm 4 \times 10^{-3}$	6.7 ± 0.3	-13.1 ± 0.07	-17.3
mean	1.06 ± 0.02	8.9 ± 1.9	-12.5 ± 0.5	-14.9 ± 1.9
PS-TTC-hairpin	$1.08 \pm 5 \times 10^{-3}$	6.6 ± 0.4	-15.0 ± 0.09	-23.8
PS-TTC-hairpin	$1.04 \pm 4 \times 10^{-3}$	7.3 ± 0.4	-15.6 ± 0.09	-25.5
PS-TTC-hairpin	$0.991 \pm 9 \times 10^{-3}$	3.9 ± 0.3	-17.5 ± 0.22	-33.2
PS-TTC-hairpin	$1.03 \pm 7 \times 10^{-3}$	9.98 ± 0.10	-14.9 ± 0.14	-22.4
mean	1.03 ± 0.03	6.9 ± 2.2	-15.8 ± 1.0	-26.2 ± 4.2

[a] Error given is the fitting error except for the mean, where the root mean square is noted.

Table S4a | Kinetic constants determined for A3A-catalyzed deamination of linear and hairpin DNA, using NMR-based assay.[a]

Name	Sequence, 5'-to-3'	k_{cat} (/s^{-1})	K_m (/μM)	k_{cat}/K_m (/s^{-1} mM^{-1})
Linear DNA	$A_2T_2CA_4$	0.30 ± 0.06	3000 ± 900	0.10 ± 0.03
TTC-hairpin	$T(GC)_2TTC(GC)_2T$	0.13 ± 0.03	31 ± 6	4.2 ± 1.3

Table S4b | K_i values of inhibitors of wild-type A3A against TTC-hairpin substrate.[a]

Name	Sequence, 5'-to-3'
FdZ-linear	2400 ± 940
TTFdZ-hairpin	117 ± 15
PS-TTFdZ-hairpin	160 ± 70

[a] See Methods for experimental details.

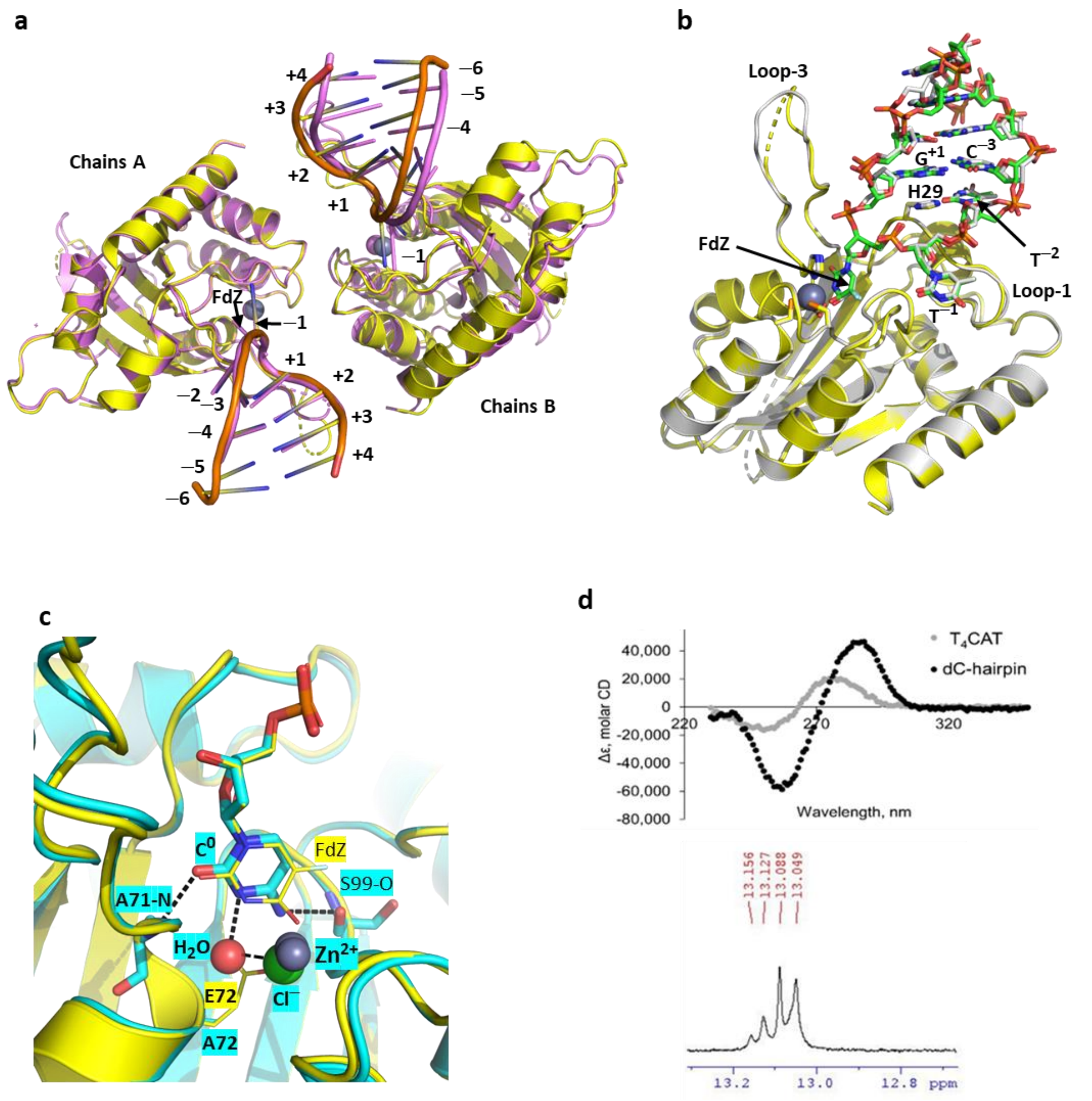

Supplementary Fig. S1 | Superpositions of structures of wildtype A3A and its inactive E72A mutant in complexes with hairpin DNA and representative proof of DNA hairpin structure in solution.

a, Superposition of subunits A of wildtype A3A complexed with TTFdZ-hairpin. The 2.94 Å resolution structure is shown in yellow (protein) and orange (hairpin); the 2.80 Å structure is shown in light purple. Note displacement and small rotation of chain B for the latter relative to the former. In general loops are better defined in chain B for both structures, whereas for the former (2.94 Å resolution) structure, the hairpin is much better defined. The Zn^{2+} is shown as grey and light purple spheres, respectively.

b, Superposition of subunit B (carbon atoms in blue) onto subunit A (carbon atoms of protein in yellow, of hairpin in green)) for the 2.94 Å resolution structure (RMSD = 0.47Å). Key active-site residues (His70, Cys101, Cys106, Glu72 and His29) are shown in stick form. Zn^{2+}

is shown as a grey sphere. Note: tight NCS restraints are placed on torsion angles of the pair of subunits. The small differences in conformations of the hairpin are not considered significant.

c, Superposition of wildtype A3A with TTFdZ-hairpin bound (yellow) and A3A-E72A with TTC-hairpin (cyan) zooming in on position 0, showing close similarity of binding and details of hydrogen bonding associated with C^0.

d, Evidence for hairpin structure in solution. Top panel: Circular dichroism spectra of linear T_4CAT and TTC-hairpin (no A3A). Bottom panel: ^{1}H NMR spectrum of TTC-hairpin in the imino region. Both spectra are consistent with formation of a hairpin structure in solution.

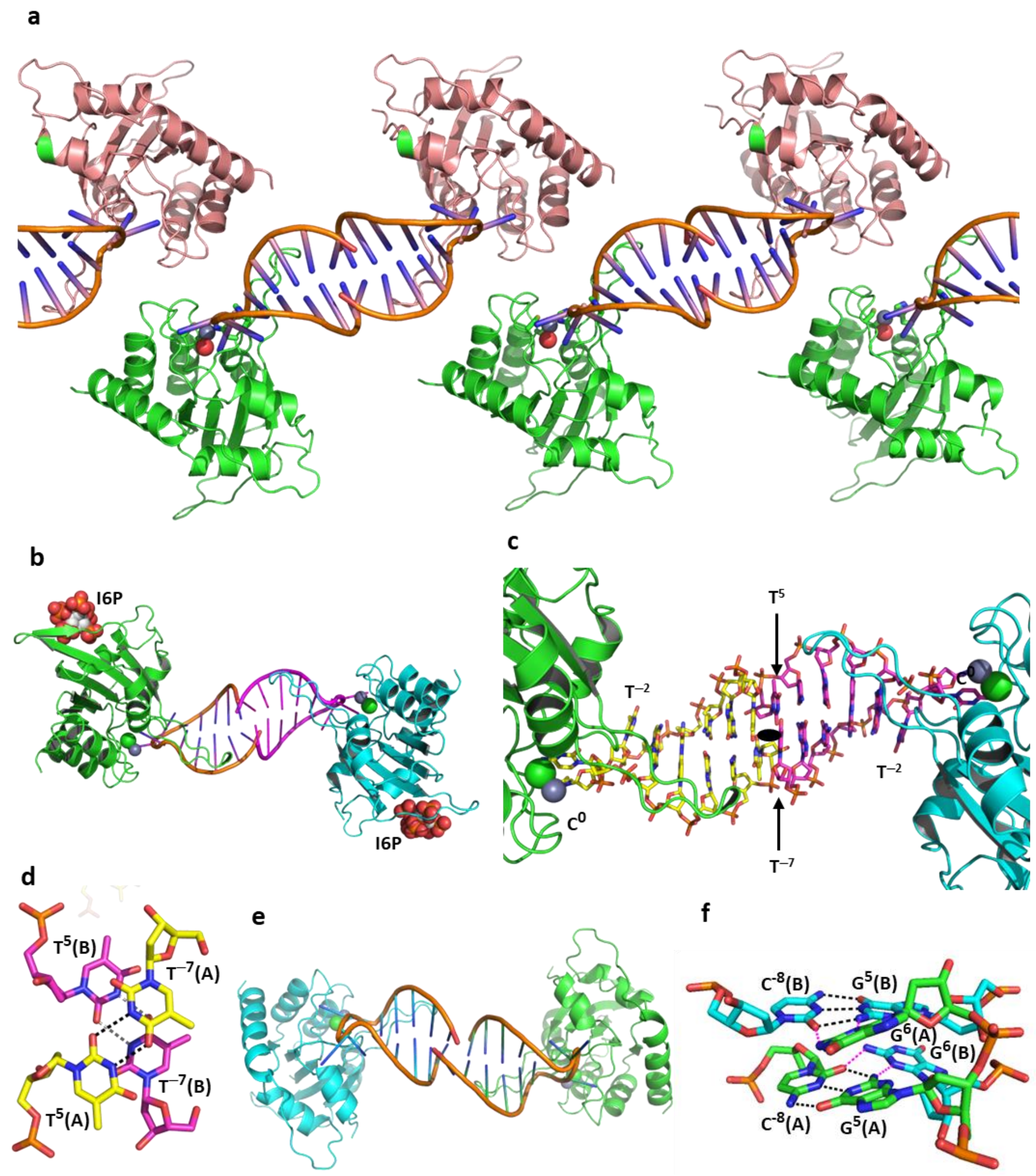

Supplementary Fig. S2 | Schematic showing the general shared packing of A3A in the unit cell and the packing of the stem bases for different hairpins.

a, In this A3A-E72A with TTC-hairpin and other structures presented here, there are two molecules in the asymmetric unit, arranged such that the base of one hairpin stacks with the base of the hairpin of the other molecule with approximate non-crystallographic two-fold symmetry. The NCS two-fold elements are shown by purple ellipses.

b, Cartoon representation of a pair of molecules for A3A-E72A in complex with TTC-hairpin. Inositol hexaphosphate (phytic acid, I6P; shown as spheres) mediates intermolecular

interactions of A3A-E72A. Stick representation detailing the interaction between the bases of the stems.

c, Stick representation showing the base pairing and projection of the TTC loop into A3A.

d, Cartoon representation of A3A-E72A in complex with ATTC hairpin showing the interaction of the base of the stem of ATTC-hairpin of one molecule with the base of the stem of another molecule. Cartoon representation of A3A-E72A in complex with ATTC-hairpin. The two-fold NCS is approximately out of the plane.

e, Cartoon representation of the interaction of ATTC-hairpin with itself and A3A-E72A.

f, Detail showing hydrogen bonding of the penultimate $C^{-8}G^{5}$ pair (black dashes) and the hydrogen bonding of the final G^{6} of one hairpin with the NCS-related $C^{-8}G^{5}$ ATTC-hairpin (magenta dashes).

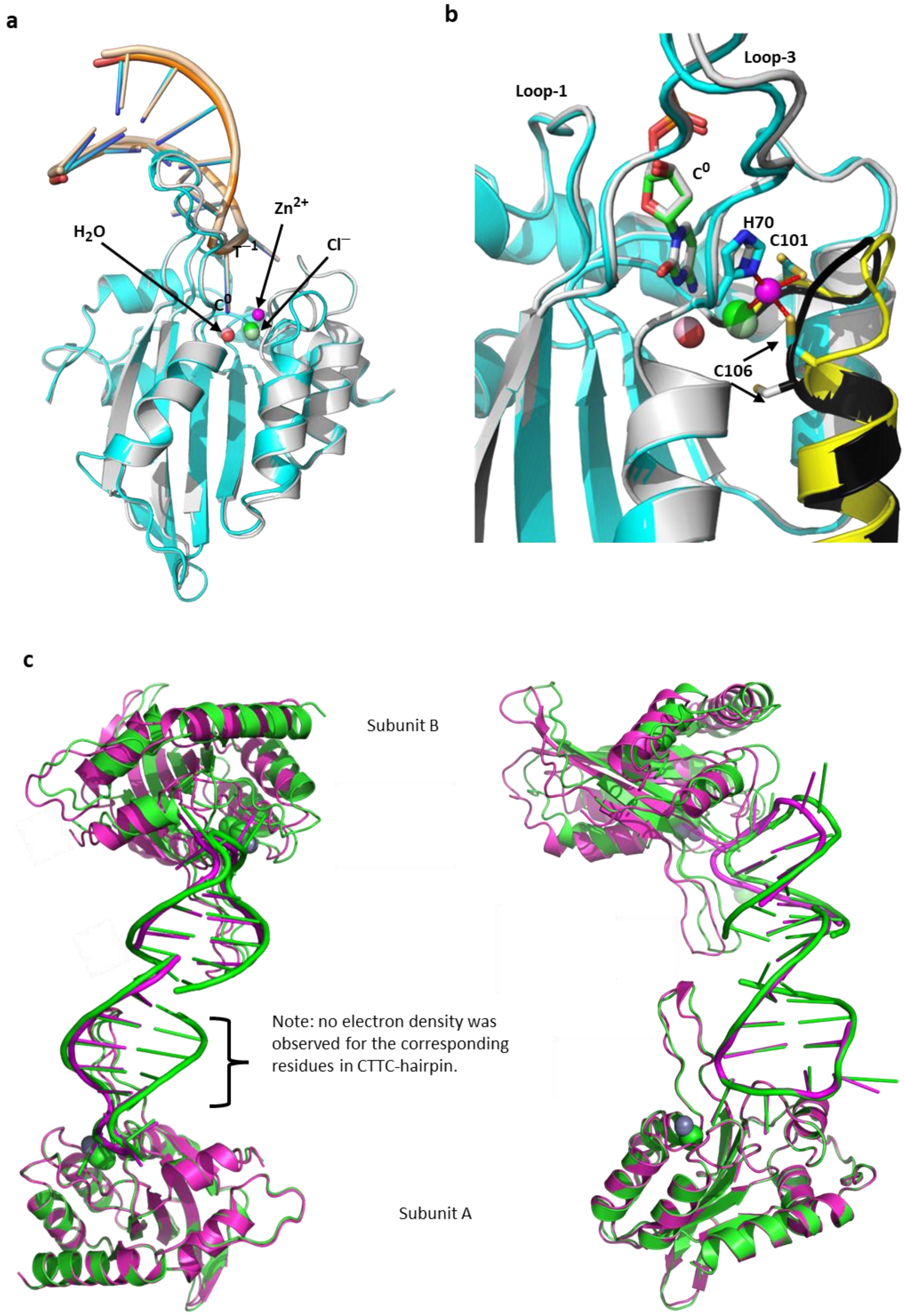

Supplementary Fig. S3 | Conservation of protein tertiary structure and hairpin conformation.

a, Superposition of structures of A3A-E72A complexed with TTC-hairpin where the Zn^{2+}-containing structure is in cyan and the Zn^{2+}-free structure is in grey. Diagram showing similarity in overall structure of protein and hairpin DNA. For the Zn^{2+}-free structure, the Cl^{-} is coloured light-green, the water light-red and the hairpin wheat.
b, Diagram of the active site showing reconfiguration of the loop bearing Cys101 and Cys106 in the absence of Zn^{2+}. The loop is highlighted in yellow for Zn^{+2}-containing structure and black for the Zn^{2+}-free structure. Cytosine C^0 is shown in sticks (green for Zn^{2+}-containing structure; grey for Zn^{2+}-free structure). Note small displacements of active site water, chloride ion and ligands His70 and Cys101, but large displacement of Cys106 on loss of Zn^{2+}.
c, Superposition of A subunits of complexes of A3A-E72A with CTTC-hairpin (magenta) onto the complex with ATTC-hairpin (green). The apparent stem bases for CTTC-hairpin are marked by magenta spheres. Note that for CTTC-hairpin, one hairpin adopts the expected threading shown in **Supplementary Table S1**, but the other hairpin appears to be threaded differently, placing C^{-3} (of **Supplementary Table S1** threading) into the active site of A3A. Left frame: the view is approximately down the two-fold NCS element. Right frame: relative to left frame, the pair of molecules has been rotated about horizontal (~30°) and vertical (~90°) axes.

.

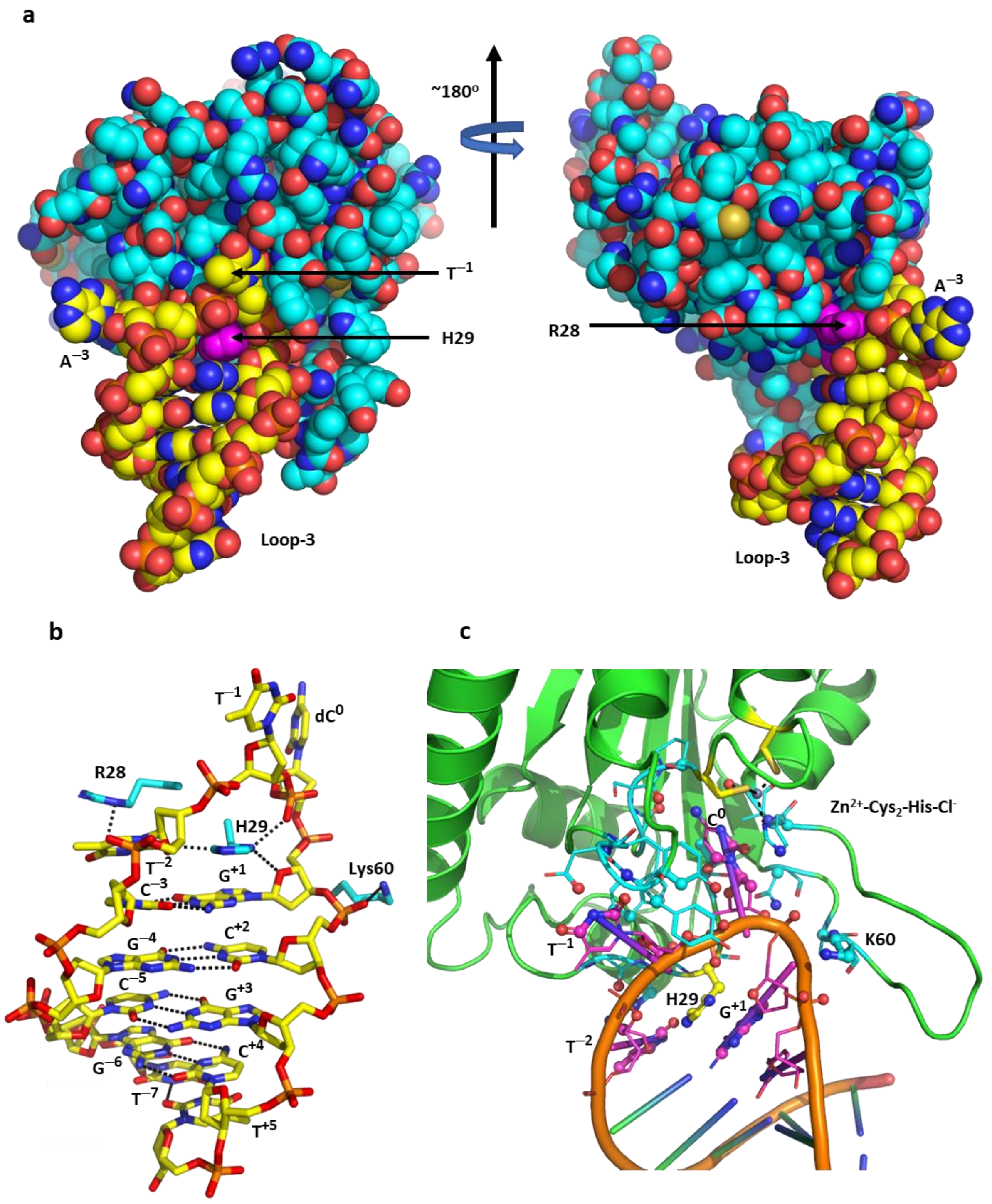

Supplementary Fig. S4 | Interactions of ATTC-hairpin with A3A-E72A.

a, Space-filling diagram of substrate/inhibitor binding to A3A, showing crucial role of His29 in the binding of substrate/inhibitor to A3A. His29 and Arg28 are highlighted in magenta; protein carbon atoms are shown in cyan and those for the ATTC-hairpin in complex with A3A-E72A in yellow; all other atoms in standard CPK colors.

b, Base-pairing in the stem of TTC-hairpin and key interactions of the hairpin with Arg28,

His29 (especially) and Lys60 (additional to interactions of dC and dT^{-1} with A3A). Similar interactions occur for ATTC- and CTTC-hairpins (less precisely) with A3A-E72A and for TTFdZ-hairpin with wildtype A3A (also less precisely).

c, Protein residues in contact with the oligo are shown in cyan for carbon atoms and oligo residues in contact with the protein are shown in magenta for carbon atoms. Atoms in contact (<3.5 Å) are shown as spheres (~40 protein and ~40 oligo atoms; ~10 residues and 4 nucleotides).

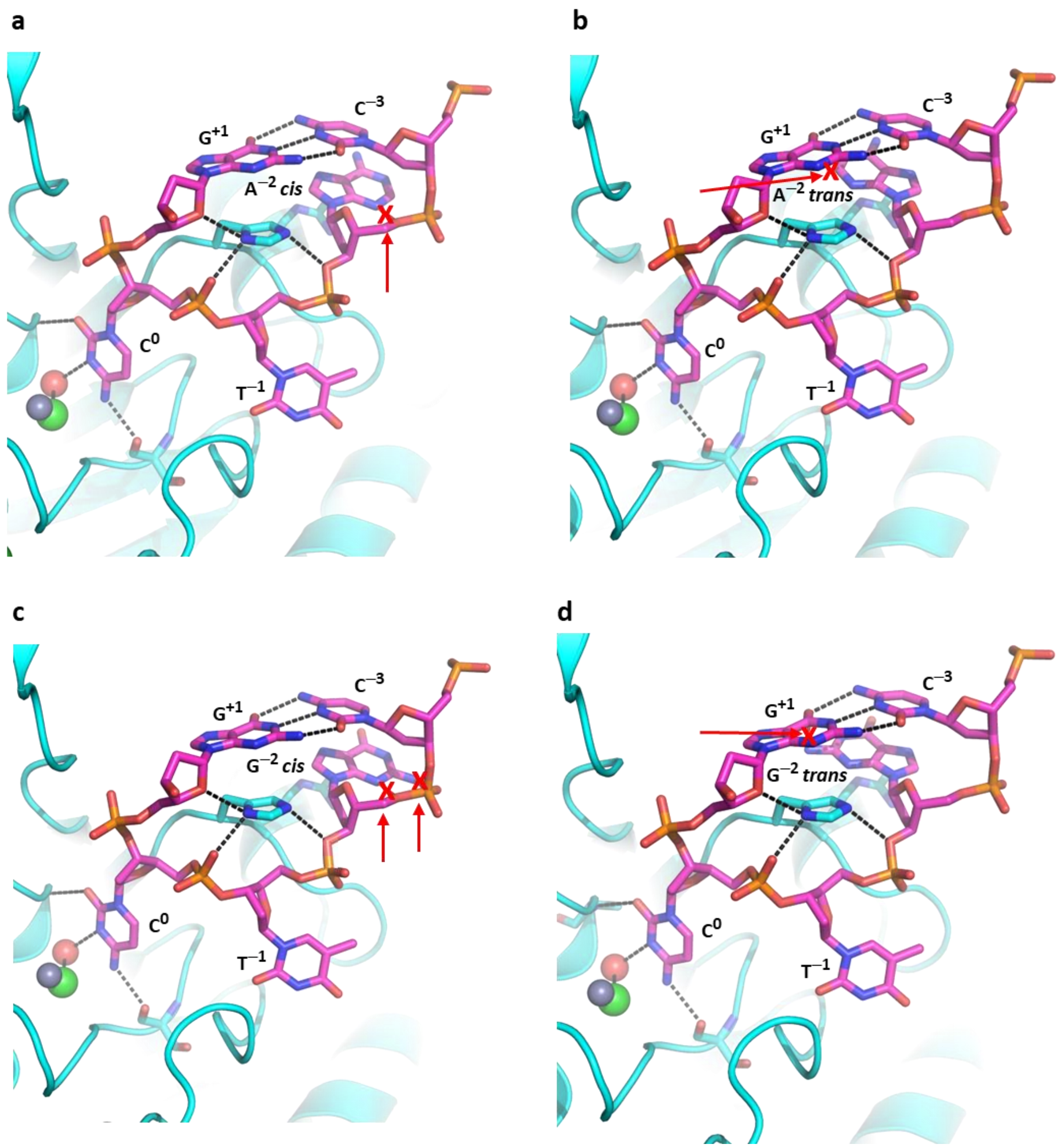

Supplementary Fig. S5 | Modelling of purines into the pyrimidine site at position −2.

a, **b**, Modelling of adenine of TTC-hairpin (magenta) in complex with A3A-E72A (cyan) in, respectively, *syn* and *anti* conformations of the glycosidic bond.

c, **d**, Modelling of guanine in, respectively, *syn* and *anti* conformations of the glycosidic bond. Clashes (< 3.1 Å) of purines in *syn* conformation with the phosphate linking nucleotides at –2 and –3 and in *anti* conformation with the nucleotide at +1 position are highlighted by red arrows and crosses. Cation-π stacking of Arg28 with purines is suboptimal compared to that with pyrimidines. Note: for the higher resolution structure of ATTC-hairpin with A3A-E72A, a water molecule (not seen for TT(C/FdZ)-hairpin structures) that bridges the carbonyl moiety at C2 of T (or C) to peptide NH of His29 cannot be accommodated for a purine in the *anti* conformation.

a

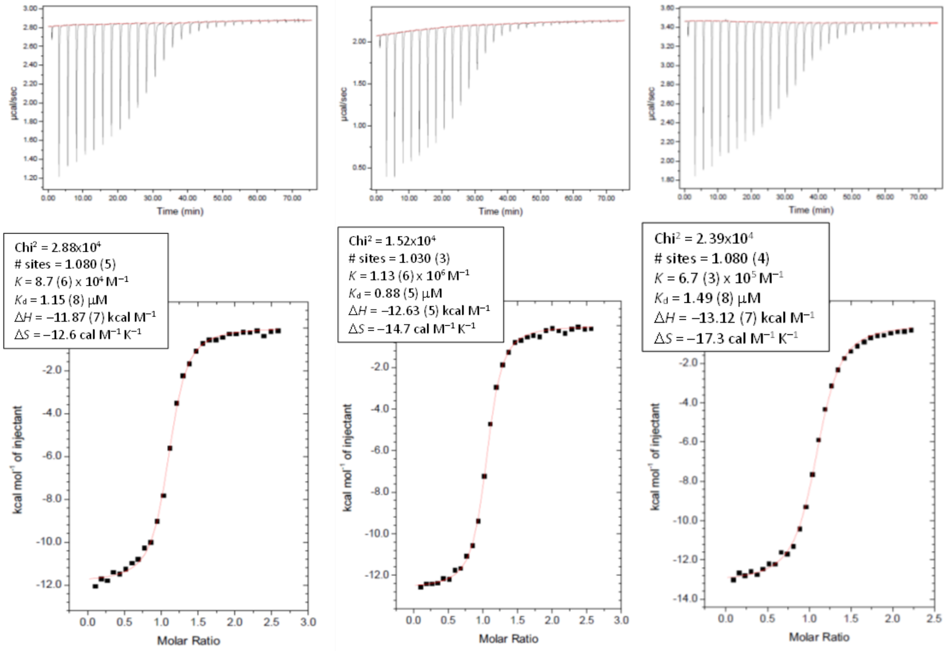

Chi^2 = 2.88x10^4
sites = 1.080 (5)
K = 8.7 (6) x 10^4 M^{-1}
K_d = 1.15 (8) µM
ΔH = −11.87 (7) kcal M^{-1}
ΔS = −12.6 cal M^{-1} K^{-1}
Chi^2 = 1.52x10^4
sites = 1.030 (3)
K = 1.13 (6) x 10^6 M^{-1}
K_d = 0.88 (5) µM
ΔH = −12.63 (5) kcal M^{-1}
ΔS = −14.7 cal M^{-1} K^{-1}
Chi^2 = 2.39x10^4
sites = 1.080 (4)
K = 6.7 (3) x 10^5 M^{-1}
K_d = 1.49 (8) µM
ΔH = −13.12 (7) kcal M^{-1}
ΔS = −17.3 cal M^{-1} K^{-1}
µcal/sec
Time (min)
kcal mol^{-1} of injectant
Molar Ratio

b

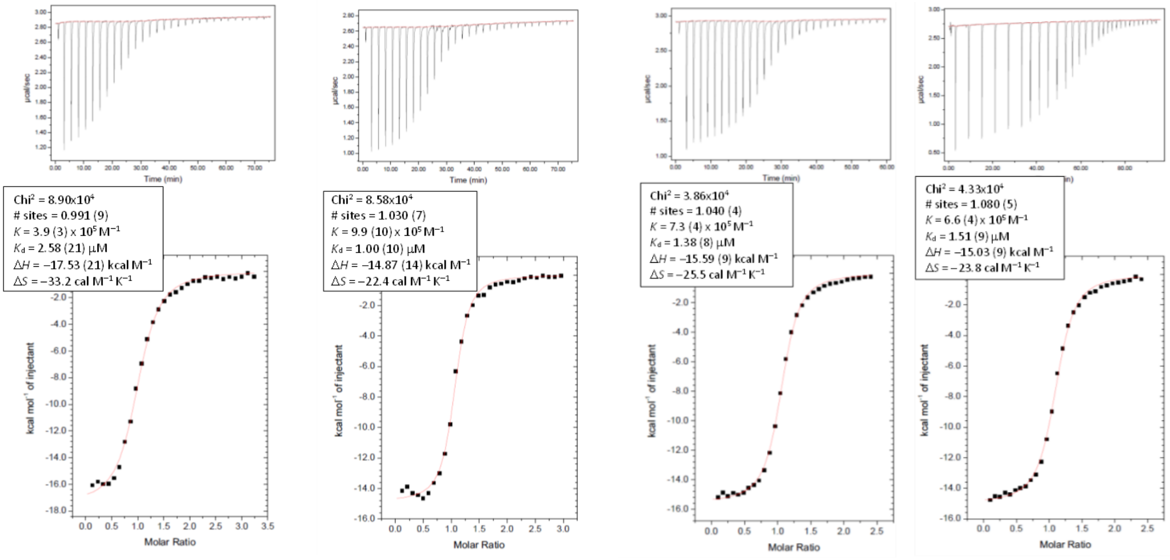

Chi^2 = 8.90x10^4
sites = 0.991 (9)
K = 3.9 (3) x 10^5 M^{-1}
K_d = 2.58 (21) µM
ΔH = −17.53 (21) kcal M^{-1}
ΔS = −33.2 cal M^{-1} K^{-1}
Chi^2 = 8.58x10^4
sites = 1.030 (7)
K = 9.9 (10) x 10^5 M^{-1}
K_d = 1.00 (10) µM
ΔH = −14.87 (14) kcal M^{-1}
ΔS = −22.4 cal M^{-1} K^{-1}
Chi^2 = 3.86x10^4
sites = 1.040 (4)
K = 7.3 (4) x 10^5 M^{-1}
K_d = 1.38 (8) µM
ΔH = −15.59 (9) kcal M^{-1}
ΔS = −25.5 cal M^{-1} K^{-1}
Chi^2 = 4.33x10^4
sites = 1.080 (5)
K = 6.6 (4) x 10^5 M^{-1}
K_d = 1.51 (9) µM
ΔH = −15.03 (9) kcal M^{-1}
ΔS = −23.8 cal M^{-1} K^{-1}
µcal/sec
Time (min)
kcal mol^{-1} of injectant
Molar Ratio

c

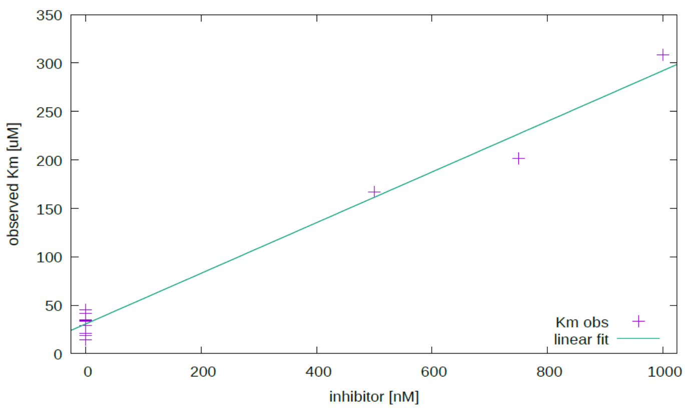

observed Km [uM]
inhibitor [nM]
Km obs
linear fit

Supplementary Fig. S6 | Isothermal titration calorimetry (ITC) data and inhibition data.

a, ITC titration of A3A-E72A with TTC-hairpin (3 replicates). See Methods for experimental conditions.

b, ITC titration of A3A-E72A with PS-TTC-hairpin (4 replicates). See Methods for experimental conditions.

c, Plot of apparent K_m *versus* inhibitor concentration for reaction of TTC-hairpin with wildtype A3A in the presence TTFdZ-hairpin inhibitor. The value of K_i is obtained from the ratio of slope/intercept of this graph.

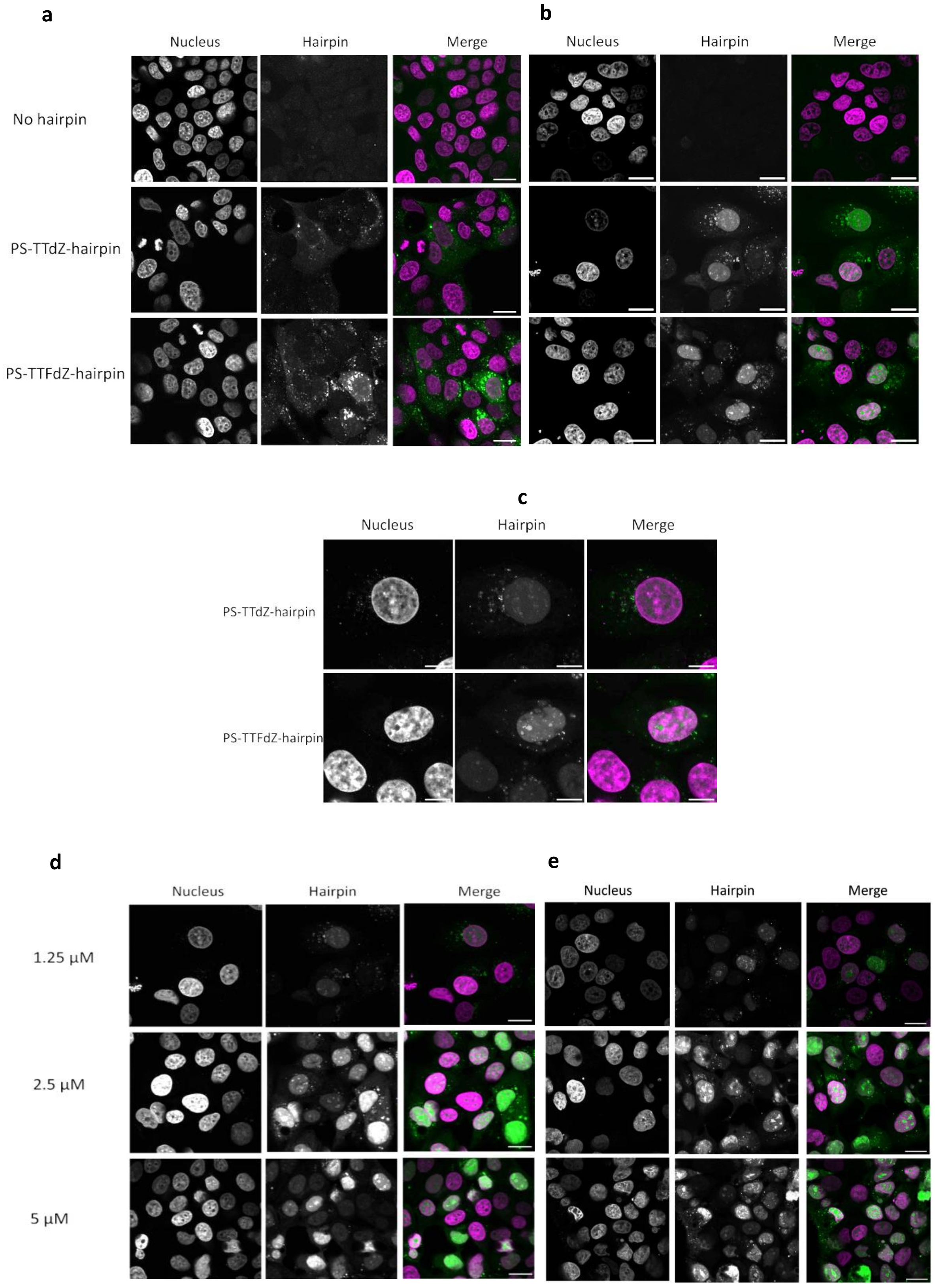

Supplementary Fig. S7 | Representative confocal microscopy images of MCF-7 cells incubated with FAM-labelled phosphorothioated- (PS-) modified hairpins at varying concentrations (0-20 µM). Hoechst 3342 is used for staining of the nucleus and the hairpin

is labelled with fluorescein (FAM) at the 3′ end. Individual panels, nucleus/Hoechst 33342 (left) and oligo/FAM (middle) are shown for each section, along with merge where pseudo-coloured panels (right) are overlaid, nucleus (magenta) and oligo-FAM (green). Except where indicated otherwise, the scale bars are 20 μm.

a, Representative images of MCF-7 cells showing absence of uptake of 20 μM PS-modified FAM-labelled hairpins (no oligo (top panels) PS-TTdZ- (middle panels) and PS-TTFdZ-hairpin (bottom panels)) *in the absence of transfection reagent after 16 hours*.

b, Representative images of MCF-7 cells incubated with no hairpin (upper panels) or 1.25 μM of PS-TTdZ- (middle panels) or PS-TTFdZ- FdZ (lower panels) hairpins *in the presence of X-tremeGENE 9 DNA Transfection Reagent after 16 hours*.

c, Zoomed images of cells shown in (**b**). Scale bars, 10 μm.

d, Representative images of MCF-7 cells incubated with PS-TTdZ-hairpin at 1.25 μM (upper panels), 2.5 μM (middle panels), 5 μM (lower panels) in the presence of transfection reagent (Xtreme GENE^{TM} HP) after incubation for 16 hours. **e**, Representative images of MCF-7 cells incubated with PS-TTFdZ-hairpin at 1.25 μM (upper panels), 2.5 μM (middle panels), 5 μM (lower panels) in the presence of transfection reagent (Xtreme GENE^{TM} HP) after incubation for 16 hours.

a

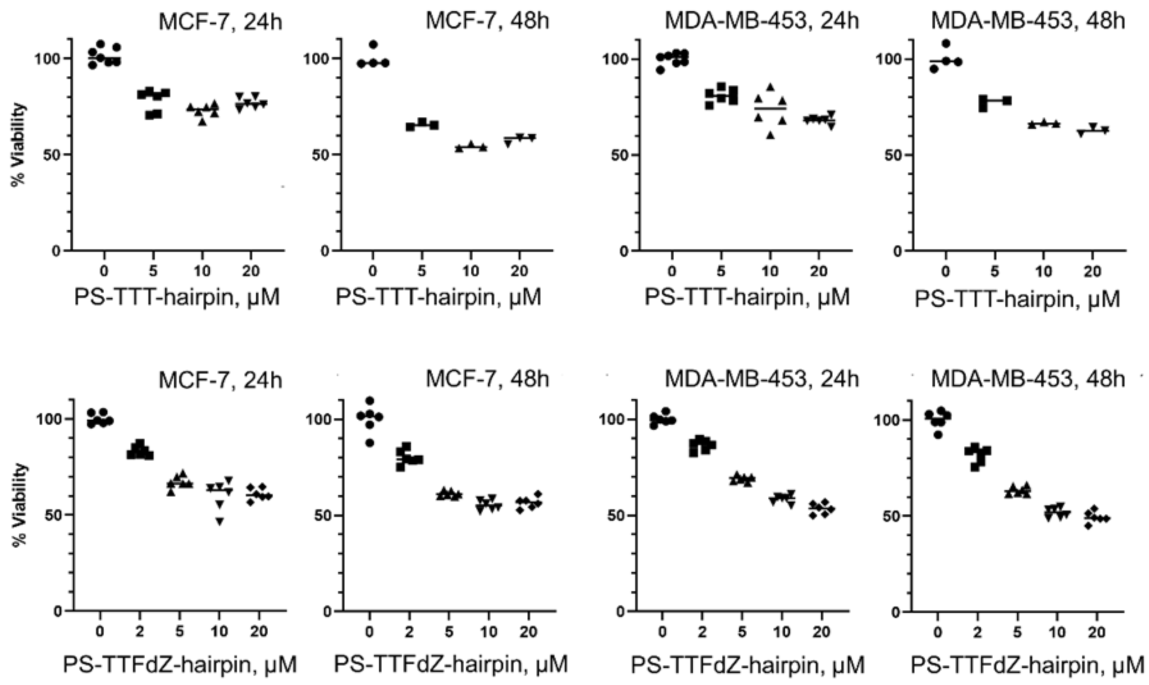

b

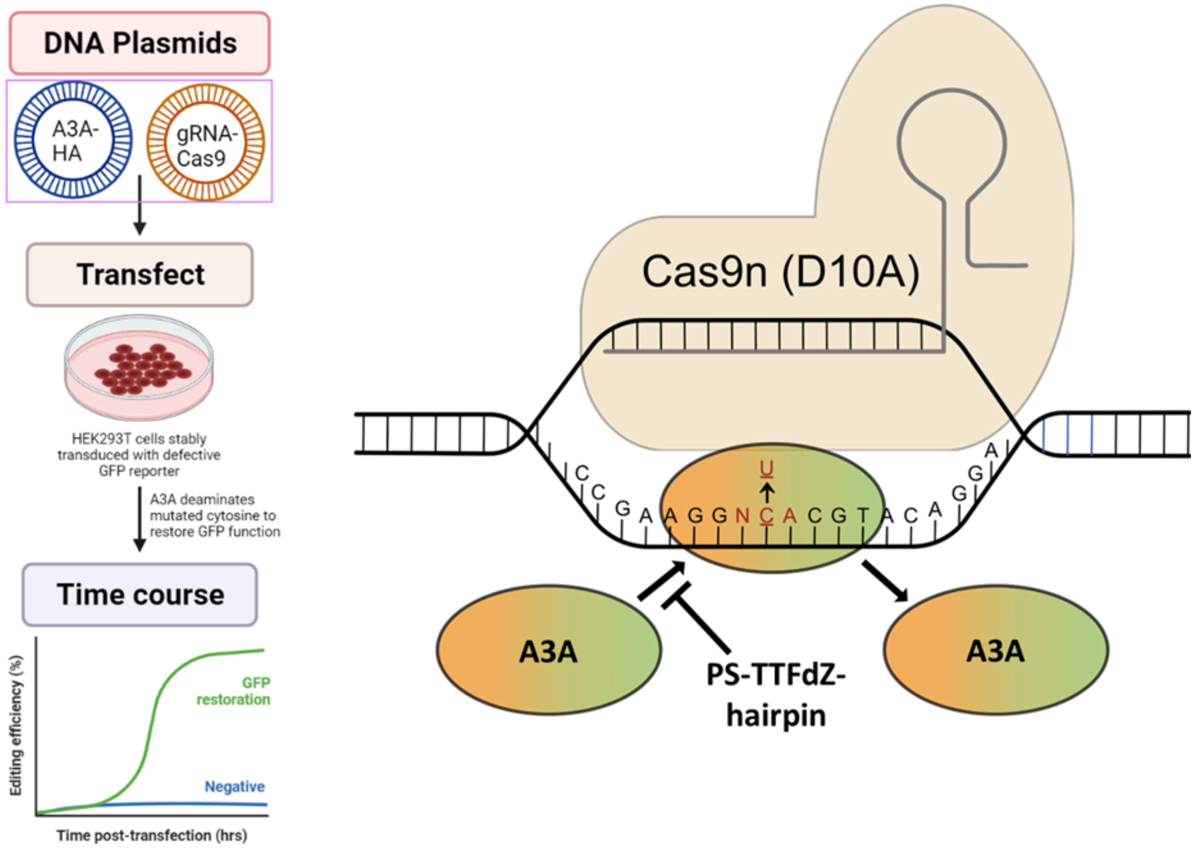

Supplementary Fig. S8 | Viability (%) of MCF-7 and MDA-MB-453 cells at various concentrations PS-modified hairpins and *in cellulo* assay of inhibitor potency.

a, PS-TTT-hairpin (upper panels) and PS-TTFdZ-hairpin (lower panels) at 24 h and 48 h, as indicated on the Figure. Data are normalized to average number of cells in the presence of transfection reagent only. Data were collected from three individual experiments with biological replicates except for the PS-TTT-hairpin at 48 h.

b, The transfection of HEK293 and expression of A3A *in cellulo*. The left schematic summarizes the experimental procedure (designed using BioRender). The right cartoon shows that the Cas9n/gRNA complex creates an R-loop and exposes the target 5’NCA trinucleotide (here 5’TCA) to C-to-U editing by untethered A3A (here unlinked to Cas9n), which restores eGFP functionality. PS-TTFdZ-hairpin inhibits A3A and therefore inhibits also the restoration of eGFP function.

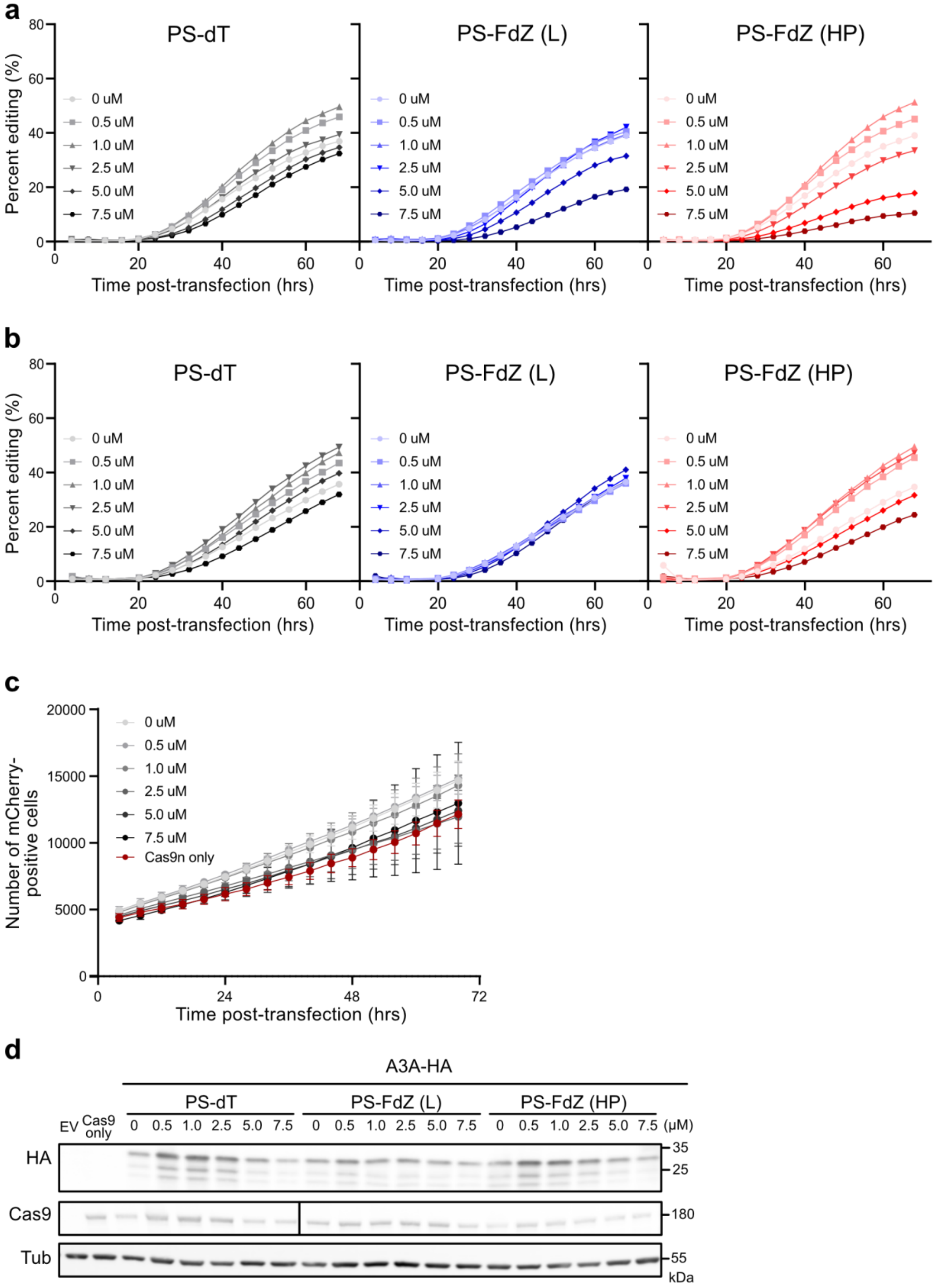
a
b
PS-dT
PS-FdZ (L)
PS-FdZ (HP)
Percent editing (%)
Time post-transfection (hrs)
0 uM
0.5 uM
1.0 uM
2.5 uM
5.0 uM
7.5 uM
c
Number of mCherry-positive cells
Cas9n only
d
A3A-HA
EV
Cas9 only
(μM)
HA
Cas9
Tub
kDa

Supplementary Fig. S9 | Experimental replicates of in cellular A3A inhibition studies.
a-b, Replicates of *in cellulo* assay of A3A-editing activity by linear and hairpin DNA. PS-TTFdZ-linear is denoted FdZ (L); PS-TTT-hairpin (as control) is denoted dT; and PS-TTFdZ-hairpin is denoted FdZ (HP) for a total of three biologically independent replicates (see Fig. 5b,c).
c, Representative example of mCherry cell count over time during live cell assay. Error bars depict mean of cell count between the three oligos tested [dT, FdZ (L), and FdZ (HP)] at each concentration. The red line represents the Cas9-only control (two technical replicates).
d, Representative immunoblots showing expression levels of A3A and Cas9 with expression of tubulin as reference, as a function of added oligonucleotide. Antibodies used are detailed in Methods. The black bar in the Cas9 panel was used to help correctly line up bands that were skewed due to curving of blot. Raw immunoblots are shown in Supplementary Fig. S10c.

a

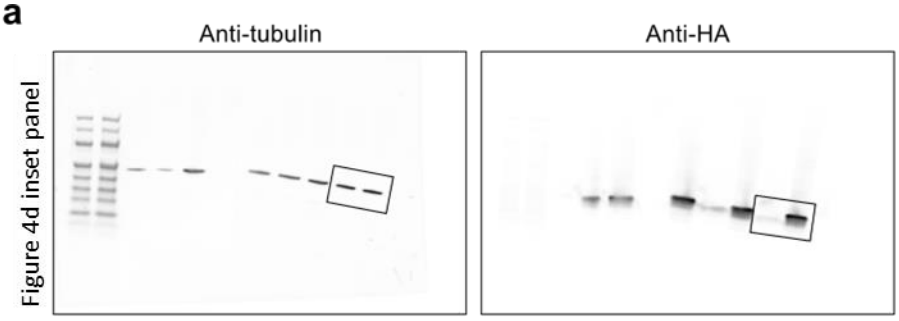
Anti-tubulin
Anti-HA
Figure 4d inset panel

b

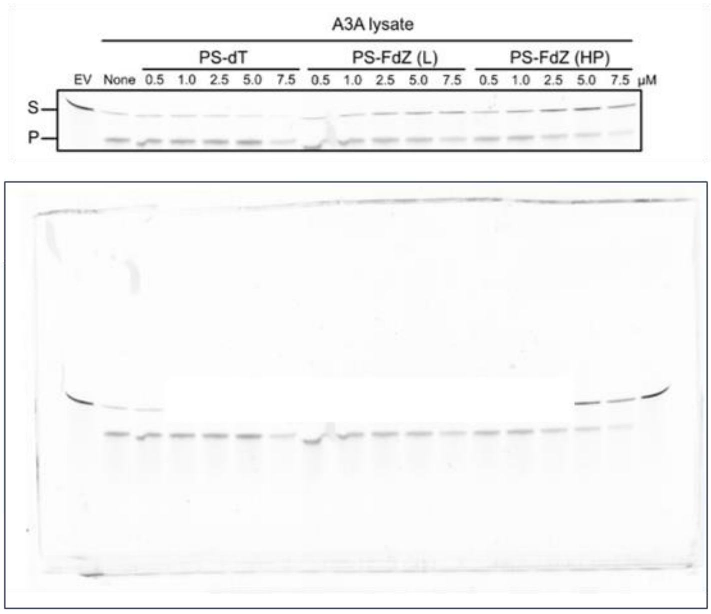
A3A lysate
PS-dT
PS-FdZ (L)
PS-FdZ (HP)
EV
None
0.5 1.0 2.5 5.0 7.5
0.5 1.0 2.5 5.0 7.5
0.5 1.0 2.5 5.0 7.5
μM
S
P

c

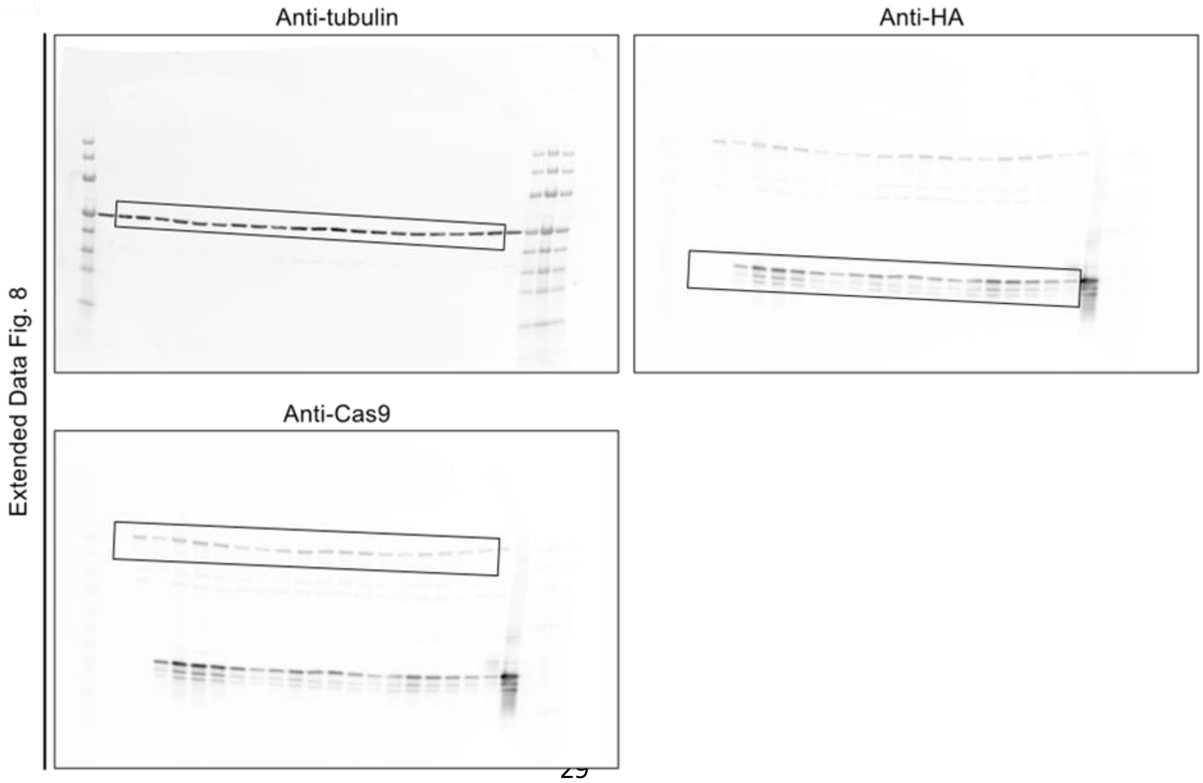
Anti-tubulin
Anti-HA
Anti-Cas9
Extended Data Fig. 8

Supplementary Fig. S10 | Original and uncropped SDS-PAGE and immunoblots.

a, Raw immunoblot images establishing expression of A3A in cell lysates. The portion of the immunoblot used for the inset panel in Fig. 4d is outlined with a black rectangle. The ladder used to mark the molecular weight is the PageRuler Prestained Protein Ladder (10 to 180kDa).

b, Cropped and labelled SDS-PAGE (upper panel) showing (i) lack of inhibition of A3A activity in cell lysates on substrate (denoted S), which is converted to product (denoted P) independent of concentration of non-inhibitor PS-dT; (ii) concentration-dependent weak inhibition by PS-FdZ (L) and strong concentration-dependent inhibition by PS-FdZ (HP). The lower panel provides an image of the complete SDS-PAGE, stained for oligonucleotides.

c, Raw immunoblots from which the cropped immunoblots in Supplementary Fig. S9d were derived.
