# Supplementary material for: Structure-guided inhibition of the cancer DNA-mutating enzyme APOBEC3A
Source: Nat Commun. 2023 Oct 11;14:6382. doi: 10.1038/s41467-023-42174-w (PMC10567711; doi:10.1038/s41467-023-42174-w)
Supplement: Supplementary file 3 — Reporting Summary [file 41467_2023_42174_MOESM3_ESM.pdf]

nature portfolio

Corresponding author(s): Geoffrey B Jameson

Last updated by author(s): Sep 6, 2023

Reporting Summary

Nature Portfolio wishes to improve the reproducibility of the work that we publish. This form provides structure for consistency and transparency in reporting. For further information on Nature Portfolio policies, see our Editorial Policies and the Editorial Policy Checklist.

Statistics

For all statistical analyses, confirm that the following items are present in the figure legend, table legend, main text, or Methods section.

n/a	Confirmed	
[ ]	[x]	The exact sample size (*n*) for each experimental group/condition, given as a discrete number and unit of measurement
[ ]	[x]	A statement on whether measurements were taken from distinct samples or whether the same sample was measured repeatedly
[x]	[ ]	The statistical test(s) used AND whether they are one- or two-sided<br>*Only common tests should be described solely by name; describe more complex techniques in the Methods section.*
[x]	[ ]	A description of all covariates tested
[x]	[ ]	A description of any assumptions or corrections, such as tests of normality and adjustment for multiple comparisons
[x]	[ ]	A full description of the statistical parameters including central tendency (e.g. means) or other basic estimates (e.g. regression coefficient) AND variation (e.g. standard deviation) or associated estimates of uncertainty (e.g. confidence intervals)
[x]	[ ]	For null hypothesis testing, the test statistic (e.g. *F*, *t*, *r*) with confidence intervals, effect sizes, degrees of freedom and *P* value noted<br>*Give P values as exact values whenever suitable.*
[x]	[ ]	For Bayesian analysis, information on the choice of priors and Markov chain Monte Carlo settings
[x]	[ ]	For hierarchical and complex designs, identification of the appropriate level for tests and full reporting of outcomes
[x]	[ ]	Estimates of effect sizes (e.g. Cohen's *d*, Pearson's *r*), indicating how they were calculated

Our web collection on statistics for biologists contains articles on many of the points above.

Software and code

Policy information about availability of computer code

Data collection	Single-crystal X-ray diffraction at the Australian Synchrotron: JBluIce-EPICS
Data analysis	X-ray data analysis: XDS, Aimless, Pointless, MolRep, Refmac5, Coot. Analysis of cellular images: ImageJ (Rasband, W. 2014. ImageJ. U.S. National Institutes of Health, Bethesda, MD). Data presentation: GraphPad Prism (Dotmatics)

For manuscripts utilizing custom algorithms or software that are central to the research but not yet described in published literature, software must be made available to editors and reviewers. We strongly encourage code deposition in a community repository (e.g. GitHub). See the Nature Portfolio guidelines for submitting code & software for further information.

Data

Policy information about availability of data

All manuscripts must include a data availability statement. This statement should provide the following information, where applicable:

- Accession codes, unique identifiers, or web links for publicly available datasets
- A description of any restrictions on data availability
- For clinical datasets or third party data, please ensure that the statement adheres to our policy

PDB codes: 8FIM (A3A-E72A with TTC-hairpin DNA substrate), 8FIL (Zn2+-free A3A-E72A with TTC-hairpin DNA substrate), 8FIK (A3A-E72A with ATTC-hairpin DNA substrate), 8FIJ (wildtype A3A with TTFdZ-hairpin inhibitor form 2), 8FII (wildtype A3A with TTFdZ-hairpin inhibitor form 1). The associated doi's are also provided

Human research participants

Policy information about studies involving human research participants and Sex and Gender in Research.

Reporting on sex and gender	*Use the terms sex (biological attribute) and gender (shaped by social and cultural circumstances) carefully in order to avoid confusing both terms. Indicate if findings apply to only one sex or gender; describe whether sex and gender were considered in study design whether sex and/or gender was determined based on self-reporting or assigned and methods used. Provide in the source data disaggregated sex and gender data where this information has been collected, and consent has been obtained for sharing of individual-level data; provide overall numbers in this Reporting Summary. Please state if this information has not been collected. Report sex- and gender-based analyses where performed, justify reasons for lack of sex- and gender-based analysis.*
Population characteristics	*Describe the covariate-relevant population characteristics of the human research participants (e.g. age, genotypic information, past and current diagnosis and treatment categories). If you filled out the behavioural & social sciences study design questions and have nothing to add here, write "See above."*
Recruitment	*Describe how participants were recruited. Outline any potential self-selection bias or other biases that may be present and how these are likely to impact results.*
Ethics oversight	*Identify the organization(s) that approved the study protocol.*

Note that full information on the approval of the study protocol must also be provided in the manuscript.

Field-specific reporting

Please select the one below that is the best fit for your research. If you are not sure, read the appropriate sections before making your selection.

☒ Life sciences ☐ Behavioural & social sciences ☐ Ecological, evolutionary & environmental sciences

For a reference copy of the document with all sections, see nature.com/documents/nr-reporting-summary-flat.pdf

Life sciences study design

All studies must disclose on these points even when the disclosure is negative.

Sample size	No statistical pre-determination of sample size calculation. Sample size was determined by sensitivity of method/instrument. Clear trends apparent as functions of time, concentration and appropriate controls and replicates validate choices of sample sizes.
Data exclusions	No data were excluded from averages presented.
Replication	Replicates were performed. Details are provided in Methods and captions to Figures. In the case of the in cellulo assays of specific inhibition of A3A gene-editing activity, variability in uptake of the inhibitor, apparent in confocal microscopy images presented, prevented averaging of replicates, which are provided in Supplementary Information; similar concentration-dependent inhibitory behaviour was observed with similar estimated IC50.
Randomization	Not applicable.
Blinding	Not applicable.

Reporting for specific materials, systems and methods

We require information from authors about some types of materials, experimental systems and methods used in many studies. Here, indicate whether each material, system or method listed is relevant to your study. If you are not sure if a list item applies to your research, read the appropriate section before selecting a response.

Materials & experimental systems

n/a	Involved in the study	
☐	☒	Antibodies
☐	☒	Eukaryotic cell lines
☒	☐	Palaeontology and archaeology
☒	☐	Animals and other organisms
☒	☐	Clinical data
☒	☐	Dual use research of concern

Methods

n/a	Involved in the study	
☒	☐	ChIP-seq
☒	☐	Flow cytometry
☒	☐	MRI-based neuroimaging

Antibodies

Antibodies used	Primary antibodies include mouse anti-Tubulin (#T5168, 1:10000, Sigma-Aldrich), rabbit anti-HA (#3724S, 1:2500, Cell Signaling), and rabbit anti-Cas9 (#ab189380, 1:5000, Abcam). Secondary antibodies used were goat anti-rabbit IRdye800 (LI-COR, #925-32211, 1:10000) and goat anti-mouse IRdye680 (LI-COR, #926-69020, 1:10000)
Validation	These antibodies are in frequent routine usage in the Harris lab.

Eukaryotic cell lines

Policy information about cell lines and Sex and Gender in Research

Cell line source(s)	MCF7 (RRID: CVCL_0031;ATCC, USA), MDA-MB-453, HEK 293T
Authentication	Authenticated at time of acquisition
Mycoplasma contamination	Cell lines tested monthly with commercial kit
Commonly misidentified lines (See ICLAC register)	The three cell lines listed above are found in the ICLAC register Table 1, but not Table 2 (authentic stock known to exist)
